# Family-Based Treatment - the Victorian roll-out: strategies, controversies and outcomes

**DOI:** 10.1186/2050-2974-1-S1-O59

**Published:** 2013-11-14

**Authors:** Martin Pradel, Claire Diffey, Michelle Roberton, Beth Shelton

**Affiliations:** 1 The Victorian Centre of Excellence in Eating Disorders, Australia

## 

What do young people and families living with an eating disorder need from a public health service system? What do clinicians and services need to provide treatment, particularly family-based treatment? How do we go about creating a public health system which provides early, equitable and quality access to evidence-based treatment for young people?

The first priority of the Victorian Centre of Excellence in Eating Disorders (CEED) is supporting the provision of family-based treatment for eating disorders across public child and youth mental health services. Inspiring change for the person (child, youth and families) and for the public health treatment context is central in CEED's work towards this priority. The engagement on the ground with metropolitan and rural services across Victoria has resulted in developing initiatives in training, case consultation and service system development.

This presentation aims to inform service leaders and clinicians about key aspects of implementing family-based treatment in public mental health services. It will provide an outline of CEED's approach to dissemination and implementation, and provide data about Victorian services utilization of family-based treatment as an evidence-based first-line treatment model. Key challenges and controversies will be canvassed.

This abstract was presented in the **Children and Youth Treatment and Service Development** stream of the 2013 ANZAED Conference.

